# Identification and Expression Analysis of *Lipoxygenase* Gene in Bitter Gourd (*Momordica charantia*)

**DOI:** 10.3390/genes15121557

**Published:** 2024-11-29

**Authors:** Haicui Ge, Shuang Liu, Hongzhe Zheng, Pengyan Chang, Weiqun Huang, Shanshan Lin, Jingyuan Zheng, Honglong Li, Zedong Huang, Qi Jia, Fenglin Zhong

**Affiliations:** 1 College of Horticulture, Fujian Agriculture and Forestry University, Fuzhou 350002, China; gehaicuifu@126.com (H.G.); liushuangsyau@163.com (S.L.); zhz19990910@163.com (H.Z.); 2 Fuzhou Smart Agriculture (Seed Industry) Industry Innovation Center, Fuzhou 350002, China; 3 Subtropical Agriculture Research Institute, Fujian Academy of Agricultural Sciences, Zhangzhou 363005, China; cpy155165@163.com; 4 Fujian Seed Station, Fuzhou 350003, China; hwq36@126.com (W.H.); fjzzzz033@163.com (S.L.); 5 Vegetable Research Institute, Hunan Academy of Agricultural Sciences, Changsha 410125, China; zhengjingyuan2004@163.com; 6 Fujian Tianmei Seed Industry Technology Co., Fuzhou 350109, China; guoxin190510@163.com (H.L.); 13459107258@163.com (Z.H.); 7 Jiuquan Institute of Agricultural Sciences Research, Jiuquan 735000, China; jq17793520924@163.com

**Keywords:** bitter gourd, *M*c*LOX*, gene expression

## Abstract

Background: Lipoxygenases (LOXs) are key enzymes in the unsaturated fatty acid oxidation reaction pathway and play an important regulatory role in the synthesis of fruit aroma volatiles. Methods: *LOX* gene family members were identified in the whole genome database of bitter gourd and analyzed bioinformatically. An RT-qPCR was used to analyze the expression differences in different tissues. Monoterpenes were determined by gas chromatography-mass spectrometry (GC-MS) technique. Results: A total of 12 *LOX* gene family members were identified in the genome. The expression of *LOX* genes varied significantly among the tissues of roots, stems, leaves, flowers, fruits, seeds and tendrils. A total of 29 monoterpenes were detected in the fruits of five different fruit colors of bitter gourd, mainly containing six types of alcohols, aldehydes, terpenes, ketones, esters and alkynes, with the highest relative content of alcohols. Conclusions: The present study provides a reference for further elucidation of the biological functions of the *LOX* gene in the synthesis pathway of aroma volatiles in bitter gourd.

## 1. Introduction

LOXs are a class of non-heme ferritins that catalyze the oxygenation of polyunsaturated fatty acids containing pentadienyl structures to form hydroperoxides in plants [1]. LOXs are widely found in mammals, plants, bacteria, fungi and algae [2]. LOXs are mainly distributed in chloroplasts and some plasma membrane tissues in the cytoplasm of plants [3]. Based on the position of the carbon atom at which LOXs bind to the carbon chain of the substrate fatty acid, LOXs can be categorized into two families, 9-LOX and 13-LOX [4]. In addition, based on the similarity of sequence structure and the presence or absence of chloroplast-targeting peptides, LOXs can be categorized into I-LOXs and II-LOXs, with I-LOXs having as much as 75% similarity and containing no chloroplast-targeting peptides, and II-LOXs having lower similarity but containing chloroplast-targeting peptides [1].

The lipoxygenase pathway is involved in a variety of plant life activities, such as growth and development, stress response and fruit flavor production [5,6,7,8]. *Arabidopsis AtLOX2* and *AtLOX6* are involved in jasmonic acid (JA) synthesis induced by leaf damage [9]. Silencing *CaLOX2* in chili plants leads to reduced JA accumulation and decreased disease resistance [10]. In the study of *Cucumis sativus*, a total of 23 *LOX* genes were identified, with 12 genes showing differential expression under abiotic stress and plant growth regulator treatment [11]. Among them, small molecule flavor actives, including volatile aldehydes, alcohols and esters produced during fatty acid metabolism, play their roles in plant growth and development by participating in the synthesis of volatile flavor compounds and the repair of damaged tissues in adversity [12]. The production of C_6_ aldehydes and corresponding alcohols in tomatoes is catalyzed by the 13-lipoxygenase TomloxC, which catalyzes the production of 13-HPOs, which constitute the major aromatic substances in tomato fruits [6,13]. The characteristic flavor of strawberries is associated with the *LOX* gene [14]. *NaLOX2* is involved in the synthesis of tobacco hexanal and some green leaf volatiles [15]. In apples, *MdLOX1a* and *MdLOX5e* were identified as candidate genes involved in the production of fruit aroma volatiles, and *MdLOX1a* was associated with the biosynthesis of characteristic apple aroma esters [16]. In melons, *CmLOX01*, *CmLOX03* and *CmLOX18* were associated with the production of fruit aroma esters [17]. Six *LOX* genes in kiwifruits, including *AdLOX3*, *AdLOX4* and *AdLOX6*, were categorized as 13-LOXs, and sequence structure analysis showed that they contain chloroplast transit peptide sequences, which were considered as possible candidate genes for the regulation of volatile compound synthesis in kiwifruits [18].

Volatiles of fruits are the key determinants of characteristic aroma and mainly include esters, alcohols, aldehydes, acids and terpenoids. Currently, studies have been conducted on the fruits of tomatoes and bell peppers [19,20]. Bitter gourds (*Momordica charantia* L.) are mainly consumed as young gourds with a refreshing bitter taste. Bitter gourd fruits contain vitamins, mineral elements, amino acids, flavonoids and bioactive substances such as saponins [21]. There are many varieties of bitter gourd, which are generally classified according to the shape of the fruit, color, surface tubercle protrusion and so on. For example, the color of the fruit can be divided into white, white-green, yellow-green, light green, green, and dark green [22,23]. Bitter gourd fruits have a unique aroma, and studies have shown that the flavor of different colored fruits is also slightly different, with differences in taste and nutrient content [24,25]. The results showed that the main volatile components of bitter gourd fruits produced in Gansu were aldehydes and alcohols, and different volatiles contributed differently to the aroma of bitter gourd fruits [26].

Aroma imparts unique flavor qualities to bitter gourd fruits and has an important impact on fruit quality. However, there are fewer studies on its aroma substance components and anabolism. Since lipoxygenase is the first key enzyme to initiate the *LOX* pathway, it was hypothesized that it might play a key role in the flavor formation of bitter melon fruit. The study of the function of metabolic enzyme genes of the lipoxygenase pathway is useful for understanding the aroma synthesis in bitter gourd. The determination of monoterpene volatiles in different bitter gourd fruit colors revealed the highest relative content of alcohols. The *LOX* genes were identified for family members, and 12 *McLOX* gene-encoded proteins were analyzed for bioinformatics and gene expression analysis. This provided a basis for studying the association between volatile substances and *LOX* genes in bitter gourd fruits.

## 2. Materials and Methods

### 2.1. Identification of the McLOX

Whole-genome data files and genome annotation files of bitter gourd were downloaded from the National Gene Bank Sequence Archiving System website (https://db.cngb.org/cnsa/download/, accessed on 14 August 2024), and Arabidopsis genome files were downloaded from the official Arabidopsis website (https://www.arabidopsis.org/, accessed on 14 August 2024). The protein sequences of the six identified *AtLOXs* in *Arabidopsis* were screened for candidate genes by comparing them with those of bitter gourd using BLASTP. Meanwhile, the hidden Markov model (HMM) of the Lyase aromatic structural domain (PF00305) specific to the *LOX* gene was downloaded from the Pfam database (http://pfam.xfam.org/, accessed on 14 August 2024). *McLOX* family members were screened from the bitter gourd genome database using the HMMER 3.0 [27] software with the screening criterion of E-value ≤ e^−5^, and redundant sequences between HMMsearch and BLASTP were removed. The InterProScan program (http://www.ebi.ac.uk/Tools/InterProScan/, accessed on 14 August 2024) was used to confirm the presence of the *LOX* structural domain and the PLAT/LH2 structural domain in the *LOX* gene sequences. Genes that contain both of these two characterized structural domains are *LOX* genes, and genes missing both the complete PLAT/LH2 structural domain and the *LOX* structural domain are in the Supplementary Table.

### 2.2. Physicochemical Analysis of McLOX Proteins

Physicochemical properties and subcellular localization prediction analyses were performed using the ExPASy (https://web.expasy.org, accessed on 22 August 2024) and Plant-mPLoc (http://www.csbio.sjtu.edu.cn/bioinf/plant-multi/, accessed on 22 August 2024). To further understand the structural features of the protein, secondary and tertiary structural analyses of the protein were performed using the online websites Prabi (https://npsa-prabi.ibcp.fr, accessed on 23 August 2024) and Swiss-model (https://swissmodel.expasy.org, accessed on 23 August 2024), respectively.

### 2.3. Phylogenetic Analysis of LOX Gene Family in Bitter Gourd

A phylogenetic tree was constructed by combining McLOX protein with four species, including *Arabidopsis thaliana*, *Solanum lycopersicum* and *Populus trichocarpa*. The evolutionary tree was then constructed by neighbor joining (NJ) using MEGA7 software with Bootstrap replications set to 1000 and other parameters defaulted. The constructed tree files were uploaded to the ITOL website (https://itol.embl.de/itol.cgi, accessed on 25 August 2024) for better presentation.

### 2.4. Gene Structure Analysis and Identification of Conserved Motifs

The gene structure and protein conserved motifs of the *LOX* gene family of bitter gourd were analyzed using the online websites NCBI (https://www.ncbi.nlm.nih.gov/Structure/cdd/wrpsb.cgi, accessed on 1 September 2024) and MEME (https://meme-suite.org, accessed on 1 September 2024), respectively. We set the number of motifs parameter to 15, and the rest of the parameters were defaults. Graphs were drawn using Tbtools software v1.098775 [28].

### 2.5. Analysis of Promoter Cis-Acting Elements

The sequence of 2000 base pairs (bp) upstream of the start codon (ATG) of the *McLOX* gene in the whole genome of bitter gourd was obtained using TBtools software. The PlantCARE database (http://bioinformatics.psb.ugent.be/webtools/plantcare/html/, accessed on 3 September 2024) was used to search for cis-elements in the promoter. The prediction results were visualized by TBtools.

### 2.6. Material Handling

The test materials were harvested from Baysha Breeding Base of Fujian Tianmei Seed Industry Science and Technology Co. Bitter gourd seedlings were raised in hole trays at day/night temperatures of 25 °C/20 °C and light/dark cycles of 16 h/8 h. Seedlings were transplanted when they reached four true leaves. The bitter gourd fruits were selected 15 days after pollination. Roots, stems, leaves, flowers, fruits, seeds and tendrils of bitter gourd were collected and 0.5 g of each was sampled for tissue expression analysis. Five colors of fruits, including white bitter gourd, white-green bitter gourd, yellow-green bitter gourd, light green bitter gourd and green bitter gourd, were picked, and 0.5 g of each sample was used for volatile monoterpenes determination. Three biological replicates were set up, and all samples were wrapped in tin foil, immediately put into liquid nitrogen and stored in an ultra-low temperature refrigerator at −80 °C for spare.

### 2.7. Expression Analysis of LOX Gene in Bitter Gourd

The Plant RNA Rapid Extraction Kit was purchased from Novozymes (Nanjing, China) Biotechnology Co. Reverse transcription cDNA was synthesized using FastKing reagent from Tiangen Biochemical Technology Co. (Beijing, China). The fluorescence quantification reagent was 2 × RealStar Fas SYBR qPCR Mix from GenStar. The quantitative primers were designed using Primer 5.0 software (Supplementary Table S1). The expression of the *McLOX* gene was verified using the bitter gourd cyclin gene *McCYP* (GenBank accession number: HQ171897) as an internal reference gene [29]. The method used to calculate the relative expression of the gene was 2^−ΔΔct^ method [30]. Three biological replicates and three technical replicates were set up.

### 2.8. Determination of Volatile Substances

Headspace solid-phase microextraction (HS-SPME) technique was used in this study. The 0.5 g sample was ground and transferred to a headspace vial with a sealed cap. The extraction head was placed in the GC-MS injector at 250 °C and resolved for 7 min, and then the Perkin Elmer Clarus SQ8T GC-MS was started to collect the data.

The GC-MS conditions were as follows: interface temperature 240 °C, electron bombardment ion source; electron energy 70 eV; ion source temperature 250 °C; acquisition range 50~550 *m*/*z*; carrier gas He (purity 99.999%); column flow rate: 1 mL/min; sampler temperature 250 °C; temperature increase program: initial temperature 50 °C, increased to 180 °C at 15 °C/min; 2 °C/min. The temperature increase program conditions were as follows: initial temperature 50 °C, increase to 180 °C at 15 °C/min, increased to 240 °C at 2 °C/min.

The plots of the determined fruit volatiles were analyzed, and each peak was searched using the NIST11 mass spectral library. A positive and negative match of more than 800 and a similarity higher than 80% were used as the basis for screening. The identified volatiles were finally characterized based on the CAS number of each component and comparison with data from the literature [31]. For quantification, the relative content of each component was calculated using peak area normalization.

## 3. Results

### 3.1. Genome-Wide Characterization of the McLOX

**Figure 1:**
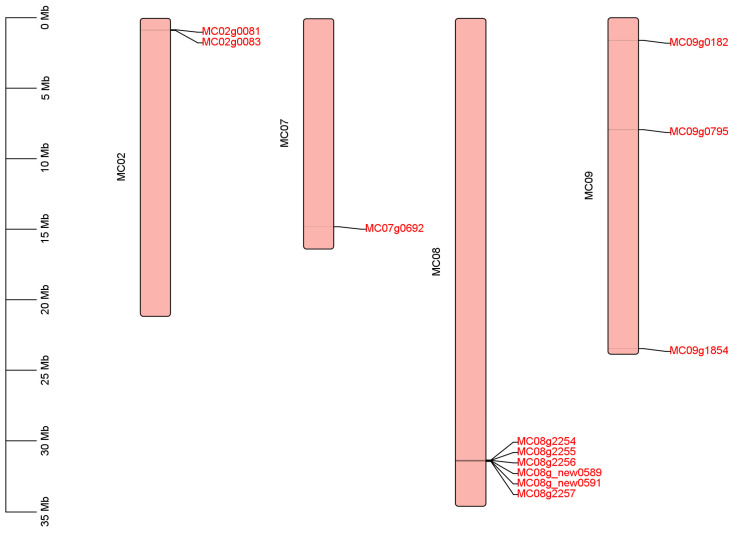
Chromosomal location of the *McLOX*.

A total of 12 *LOX* genes were identified in bitter gourd using bioinformatics (Figure 1), unevenly distributed on four chromosomes, with two genes on chromosome Mc02, only one gene on chromosome Mc07, six genes on chromosome Mc08 and three genes on chromosome Mc09. According to the position on the chromosomes, they were named *McLOX1*–*McLOX12*.

### 3.2. Physicochemical Characterization of the McLOX Gene

The physicochemical properties of the proteins of the 12 identified bitter gourd *LOX* genes were analyzed (Table 1). The number of amino acids of the proteins encoded by the family members ranged from 763 to 958; the molecular weights ranged from 86.74 to 109.65 kD. Except for three proteins, McLOX3, McLOX11 and McLOX12, which had isoelectric points greater than 7, the theoretical pIs of the rest of the proteins were less than 7, suggesting that most of the bitter gourd LOX proteins were acidic proteins. All McLOX proteins have hydrophilic coefficients less than 0 and are hydrophilic. None of the McLOX proteins have signal peptides and are non-secretory proteins. With the exception of McLOX1 and McLOX5, the other members of the family are unstable proteins with stability coefficients greater than 40. Subcellular localization prediction showed that all McLOX family members are localized to the cytoplasm.

### 3.3. Secondary and Tertiary Structure Analysis of McLOX

**Figure 2:**
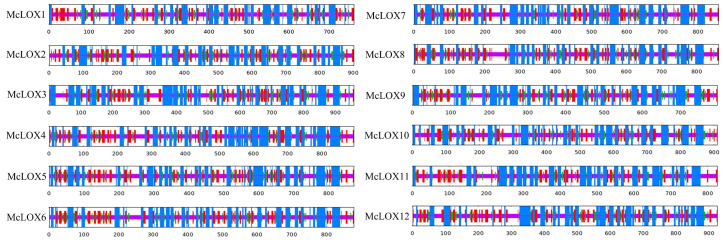
Analysis of protein secondary structure of bitter gourd *LOX* gene family.

**Figure 3:**
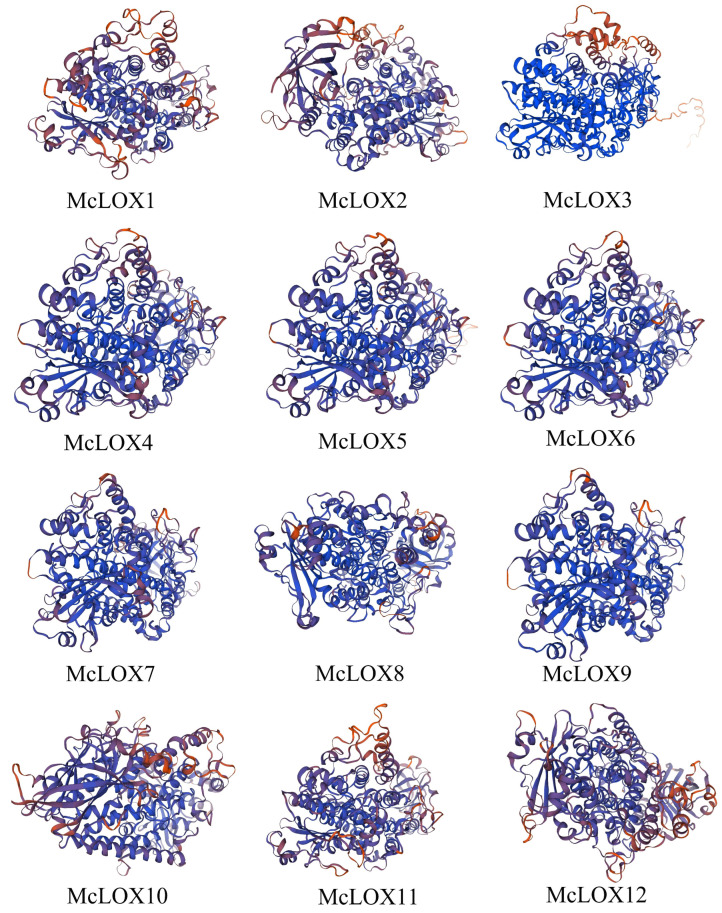
Tertiary structure of bitter gourd *McLOX*.

The sequence structure of a protein reflects the function of the protein and lays the foundation for the tertiary structure pattern of the protein. The secondary structure of the bitter gourd LOX protein was predicted (Table 2). The α-helix and irregular coil occupied the largest proportions of 31.59%–39.44% and 34.97%–38.9%, respectively. Next in order were extended strands (18.21%–20.71%) and β-turns (8.03%–11.14%) (Figure 2). The tertiary structure of bitter gourd LOX proteins was further analyzed (Figure 3), and the results showed that, except for McLOX11, which used 3pzw.1.A as a template, the rest of McLOX proteins had 8i6y.1.A as a template.

### 3.4. Phylogenetic Analysis

**Figure 4:**
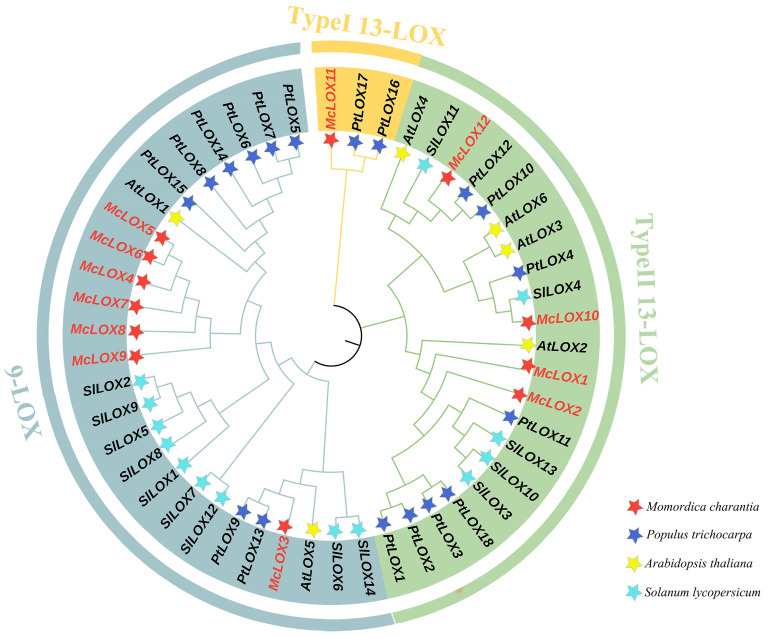
Phylogenetic tree analysis of bitter gourd McLOX proteins and LOX proteins from *A. thaliana*, *S. lycopersicum* and *P. trichocarpa*.

In order to clarify the phylogenetic relationship of LOX genes in bitter gourds, this study analyzed the phylogeny of LOX proteins from bitter gourds with *A. thaliana*, *S. lycopersicum* and *P. trichocarpa* (Figure 4). LOX proteins can be categorized into the subfamily 9-LOX and the subfamily 13-LOX, and 13-LOX proteins are further classified into two types, type I and type II, based on the protein structure; type I 13-LOX proteins lack plastid-targeting peptides and have more than 75% sequence similarity, while type II 13-LOX proteins have plastid-targeting peptides but have lower sequence similarity.

Phylogenetic analyses showed that bitter gourd and poplar genes were classified into three categories, whereas Arabidopsis and tomatoes were clustered only in the subfamily branches of 9-LOX and 13-LOXII. *McLOX3-9* belongs to the 9-LOX subfamily and is clustered with *AtLOX1* and *AtLOX5* of Arabidopsis. The type I 13-LOX subfamily has fewer members and contains only *McLOX11* of bitter gourd and *PtLOX16* and *PtLOX17* of poplar. Four genes, *McLOX1, McLOX2, McLOX10* and *McLOX12* of bitter gourd, are clustered into the type II 13-LOX subfamily, clustered with *AtLOX2-4* and *AtLOX6* of Arabidopsis.

### 3.5. Gene Structure and Conserved Motifs Analysis of McLOX

**Figure 5:**
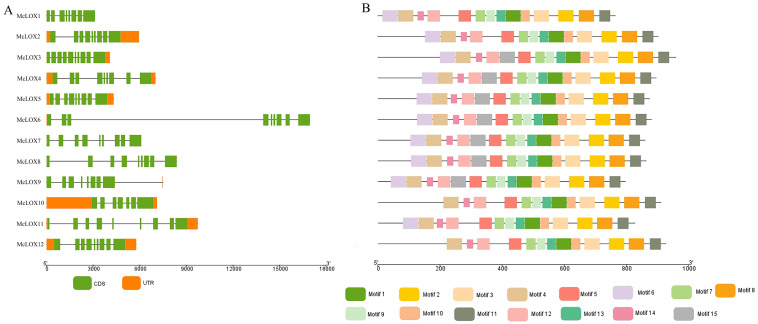
Distribution of conserved motifs and gene structure of *McLOX*. (**A**) Gene structure. (**B**) Protein conserved motifs.

All 12 McLO*X* protein sequences identified contained the complete PLAT/LH2 (IPR036392) motif, the lipoxygenase structural domain (IPR036226) and a crucial iron binding site (IPR020833) (Table 3). Intron and exon analyses were performed on the members of the bitter gourd *LOX* gene family (Figure 5A). The results showed that, except for the *McLOX1, McLOX6, McLOX7* and *McLOX8* genes, which had no UTR region, the rest of *the McLOX* genes consisted of a CDS region and a UTR region. The CDS region of *McLOX* genes was segmented by 6–9 introns, except for *McLOX1, McLOX3, McLOX9* and *McLOX10* genes, which were all segmented by eight introns, suggesting functional differentiation during the evolutionary process.

The MEME online software (https://meme-suite.org, accessed on 1 September 2024) was used for the protein conserved motif analysis of LOX protein in bitter gourd (Figure 5B), and the conserved motifs of motif1–motif15 are shown in Table 4. The results showed that bitter gourd LOX proteins contain 13–15 motifs, while McLOX1, McLOX2 and McLOX11 contain 14 motif modules and lack motif15. McLOX10 and McLOX12 contain 13 motif modules and lack motif6 and motif15. McLOX3–McLOX9 contain motif 1–motif15, indicating that these seven motif modules are highly conserved.

### 3.6. Analysis of Cis-Acting Elements in Promoters of the LOX Gene Family in Bitter Gourd

**Figure 6:**
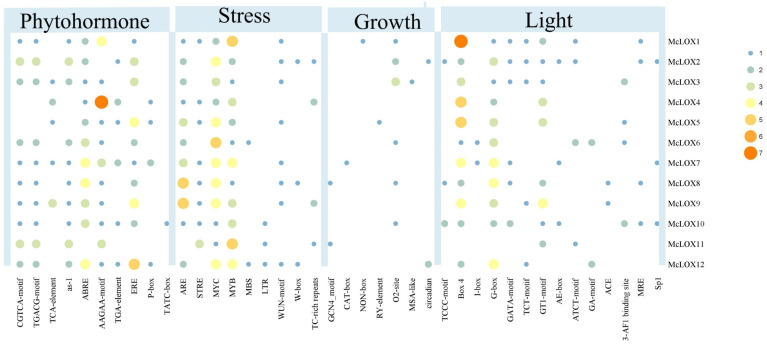
Type and number of cis-regulatory elements in the promoter of McLOX.

A 2000 bp sequence upstream of the start codon ATG of bitter gourd *LOX* gene family members was obtained for the prediction of promoter transcription start sites. A total of 433 cis-acting elements were identified (Figure 6). A total of 131 stress-associated response regulators were identified, among which MYC, ARE and W-box were more frequent. Among them, *McLOX10*, *McLOX11* and *McLOX12* genes were related to low temperature, and *McLOX6* and *McLOX12* were related to drought. A total of 14 light-related response elements, including G-box, GT1-motif, Box 4 and GATA-motif. Each of the *McLOX* genes contained light-responsive elements. A total of 10 hormone-responsive elements were identified. A total of 25 salicylic acid-responsive regulatory elements (TCA-element and as-1), 33 gibberellin-responsive regulatory elements (ERE, P-box, and TATC-box) and 8 growth hormone-responsive elements (TGA-element). The methyl jasmonate and abscisic acid response elements were more numerous, including CGTCA-motif, TGACG-motif, ABRE and AAGAA-motif. *McLOX* genes were associated with plant growth, and the response elements were GCN4-motif, CAT-box, NON-box, RY-element, O2-site, MSA-like and circadian. *McLOX* genes all contain different cis-acting elements, suggesting possible involvement in different functions in plants.

### 3.7. Analysis of Expression Patterns in Different Tissues of the LOX Gene Family in Bitter Gourd

**Figure 7:**
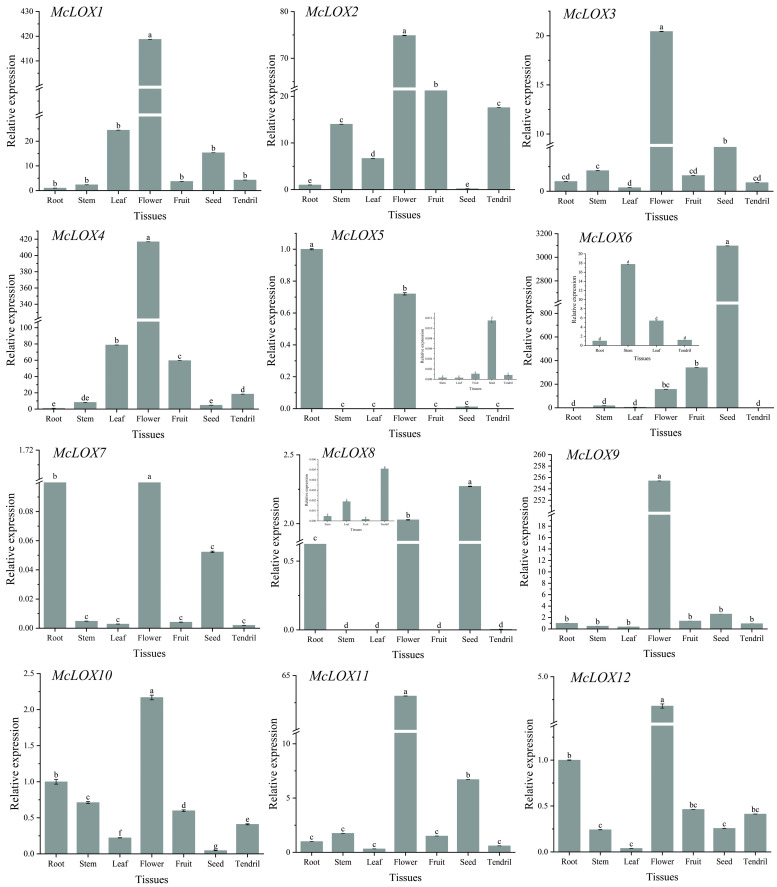
Expression pattern of *McLOX* gene in different tissue sites in bitter gourd. Error bars represent the standard errors (SEs). Different lowercase letters represent significant differences (*p* < 0.05).

In this study, cDNAs from the root, stem, leaf, flower, fruit, seed and tendril tissue sites of bitter gourd were used as templates to investigate the specific expression of the bitter gourd *McLOX* gene in different tissues. The results showed (Figure 7) that members of the *LOX* gene family in bitter gourd were expressed differently in different tissues. The expression was relatively low in stems, leaves and tendrils. Most of the genes were most highly expressed in flowers, including the *McLOX1*, *McLOX2*, *McLOX3*, *McLOX4*, *McLOX9*, *McLOX10*, *McLOX11* and *McLOX12* genes, which were expressed 418.77, 74.89, 20.44, 416.84, 255.41, 2.17, 63.12 and 4.68 times. Secondly, the expression was relatively high in fruits, especially in *McLOX2* and *McLOX4* genes, which were 54.95 and 59.58 times higher compared to roots. This indicates that the expression of genes related to the synthesis of volatile monoterpene substances would be higher in the flowers and fruits of bitter gourd than in other tissue sites. The expression of bitter gourd *McLOX5* and *McLOX7* genes was higher in roots, followed by flowers, and very little expression was found in tissue parts such as stems, leaves, fruits and tendrils. The expression of bitter gourd *McLOX6* and *McLOX8* genes was higher in seeds, which were 3097.02 and 2.27 times higher than that in roots, respectively. This indicates that *McLOX* genes have different expression patterns in different tissue parts of bitter gourd, which presumably may affect the synthesis of volatile substances.

### 3.8. Analysis of Volatile Monoterpene Content of Bitter Gourd with Different Fruit Colors

**Figure 8:**
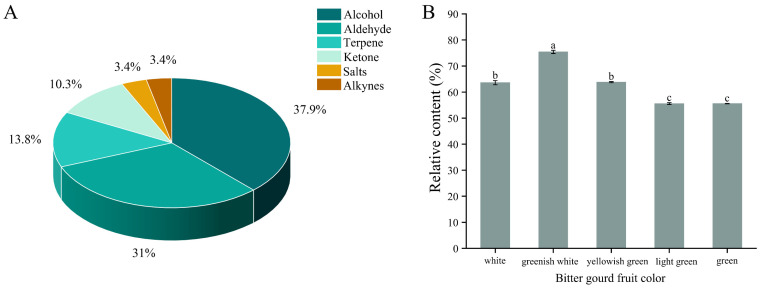
Types and relative contents of volatile monoterpenes in bitter gourd of different fruit colors. (**A**) Volatile monoterpene species. (**B**) Relative content of volatile monoterpenes per fruit color. Error bars represent the standard errors (SEs). Different lowercase letters represent significant differences (*p* < 0.05).

The volatile monoterpenes of white bitter gourd, white-green bitter gourd, yellow-green bitter gourd, light green bitter gourd and green bitter gourd fruits were determined by the GC-MS technique. A total of 29 volatile monoterpenes were detected in the fruits (Figure 8A), which mainly contained six categories, including alcohols, aldehydes, terpenes, ketones, esters and alkynes, which accounted for 37.93%, 31.03%, 13.79%, 10.34%, 3.45% and 3.45% of the total categories, respectively. Alcohols and aldehydes had the most categories, indicating that alcohols and aldehydes were more abundant in the monoterpenes of bitter gourd fruit.

As can be seen in Figure 8B, white-green bitter gourd had the highest relative content of more than 70% and more than 60% of alcohols among the five colors of fruits. White and yellow-green bitter gourd had 60% relative content of volatile monoterpenes and more than 50% of alcohols. The relative content of light green and green bitter gourd was lower but also reached more than 40%. This indicates that the relative content of alcohols in bitter gourd fruits is the highest, and it is hypothesized that the “green flavor” of bitter gourd fruits mainly comes from alcohols.

## 4. Discussion

Most of the volatile substances in fruits are synthesized through the fatty acid pathway, and lipoxygenase, as a key enzyme in the oxidation reaction of unsaturated fatty acids, plays a very important regulatory role. Some of the lipoxides produced during fatty acid metabolism, which are important for fruit flavor, are mainly produced under the catalytic effect of lipoxygenase. It was found that the components indicative of fresh tomato flavor were hexanal and hexanol, and their contents were related to tomato freshness [32]. Exogenous LOX had no significant effect on the synthesis of postharvest flavor substances in tomatoes, whereas the addition of the LOX precursor linolenic acid significantly increased the content of hexanol and hexanal in the fruit [33]. The LOX pathway plays a key role in the formation of flavor substances in tomatoes. In cucumbers, the main characteristic aromatic substance was trans-cis-2,6-nonadienal catalyzed by 9-LOX [34]. In apples, strawberries and pears, LOX was also involved in the production of flavor substances [35,36,37]. A study on the factors affecting the production of flavor substances during the postharvest ripening of prunes found that the level of LOX activity affected the content of major flavor substances in prunes [38]. The LOX pathway holds a pivotal role in the formation of fruit flavor substances.

The results of the phylogenetic analysis showed that bitter gourd and poplar genes were divided into three categories, and the *AtLOX* genes in *Arabidopsis* belonged to the 9-LOX and type II 13-LOX subfamilies [39]. In the 9-LOX subfamily, *McLOX4–9* cluster with *AtLOX1*, and *McLOX3* clusters with *AtLOX5*. The expression of *AtLOX1* is affected by pathogen infestation, abscisic acid (ABA) and methyl jasmonate (MeJA), and *AtLOX2* plays a specific role in the biosynthesis of JA precursors [39,40,41]. Therefore, it can be hypothesized that MeJA induces the expression of the *McLOX4–9* genes in bitter gourd. Subcellular predictions for members of the bitter gourd *LOX* gene family are localized in the cytoplasm. The vast majority of *Glycine max LOX* gene members are localized in the cytoplasm, which is consistent with the results of this study [42]. Most *LOX* are localized in the cytoplasm, but a large number of *LOX* genes have been detected in the spinach chloroplast envelope [43]. Different *LOXs* may have specific subcellular localization to function accordingly in different branches of the LOX pathway [44].

In the prediction of cis-acting elements of the *McLOX* gene promoter, the main cis-acting functions include four categories: abiotic stress, growth and development, hormone response and light response. The light response has the highest number of cis-acting elements. The study of buckwheat Lox gene expression patterns in response to light found that red and blue light induced the expression of *FtLox1* and *FtLox7* genes and suppressed the expression of *FtLox4* and *FtLox6* genes [45]. Phytohormones play an important role in regulating growth and development and response to adversity stress [46]. Aroma is one of the important quality characteristics of fruit, and its synthesis cannot be regulated without hormones. The topical application of MeJA on vines and leaves significantly enhances the aroma of grape berries and promotes the synthesis of bioactive substances [47]. In Picea abies, MeJA and ABA can upregulate *TcLox1* gene expression, and ABA can upregulate *TcLox2* gene expression [48]. Exogenous ethylene was able to inhibit the expression of kiwifruit *AdLox2, AdLox3* and *AdLox4* genes and reduce their expression levels [18]. In this study, the promoter region of the bitter gourd gene was found to contain hormone-responsive elements such as MeJA, salicylic acid, ABA, growth hormone and gibberellin, and it was hypothesized that the bitter gourd *LOX* gene might be induced by hormones. However, relatively few studies have been conducted on the primary genes through which hormones regulate the metabolism of aroma substances, and further related research work is needed.

*LOX* genes are widely distributed in plants with different specific functions in different parts. The fluorescence quantification of *LOX* genes in different tissues of bitter gourd was analyzed, and the results showed that the *LOX* gene family members were differentially expressed in tissue. Most *LOX* genes were most highly expressed in flowers, followed by those in fruits, especially *McLOX2* and *McLOX4* genes. Studies have shown that maize *ZmLOX* genes are expressed differently in different developmental stages and tissues, *ZmLOX4* is expressed in the root and apical meristem of maize plants and is associated with drought resistance [49]. *ZmLOX5* is expressed in the aboveground organs of maize plants, especially in the filamentous parts, and is associated with resistance to sticky insects [50]. *ZmLOX6* is expressed in chloroplasts and is associated with the regulation of pathogen infestation. *ZmLOX7* is expressed in the chloroplasts of maize plants [51], *ZmLOX8* is expressed in the chloroplasts of maize plants and *ZmLOX10* is expressed in non-chloroplast organelles of maize leaves and is associated with insect resistance and regulated by *ZmLOX8* [52]. *ZmLOX8* is expressed in chloroplasts of maize plants, and *ZmLOX10* is expressed in non-chloroplast organelles in maize leaves and is associated with insect resistance and regulated by *ZmLOX8* [53]. Currently, several *LOX* genes have been identified in apples, and their functions have been initially investigated. In the “Golden Crown” genome database, 23 *LOX* candidate genes were screened based on sequence similarity. *MdLOX2* and *MdLOX5* genes were expressed in fruit, leaf and flower tissues, while other *LOX* genes were differently expressed in the tissues [17]. Of the 22 *LOX* genes in “Jonagold” apples, 17 were expressed in the pericarp [54]. This indicates that the *LOX* gene family members were differentially expressed in tissue.

The determination and analysis of monoterpene volatiles in bitter gourd of different fruit colors showed that the relative content of monoterpenes was the highest in white-green bitter gourd, and the relative content of alcohols also accounted for the highest proportion. It was hypothesized that the “green flavor” of bitter gourd is mainly derived from alcohols. Monoterpenes have an important influence on the aroma and flavor quality of the fruit, mainly in *Malus pumila* [55], *Prunus persica* [56], *Citrus reticulata* [57] and other important flavor substances in plants, and they are widely used in food, agriculture and medicine [58,59]. The research results showed that the volatile substances of bitter gourd fruit are mainly alcohols and aldehydes, which have an important contributing role to the aroma of bitter gourd fruit [26].

Lipoxygenase is the first key enzyme that initiates the LOX pathway and is importantly linked to the synthesis of volatile substances in bitter gourd. The expression of the *LOX* gene is relatively high in bitter gourd fruits. The involvement of the *LOX* gene in the biosynthesis of C6 aldehyde volatiles has been demonstrated in *Cucumis melo*, *S*. *lycopersicum* and *G*. *max* [6]. It was found that the expression of *PpLOX2* and *PpLOX3* was reduced during peach fruit ripening, and the concentrations of hexanal and (E)-2-hexenol were decreased [60]. In kiwifruits, the expression of *AdLOX2*, *AdLOX3*, *AdLOX4* and *AdLOX6* was down-regulated during ripening, and a decrease in aldehyde correlated with reduced *LOX* expression levels [61]. In pears, the *LOX* gene expression levels were low during early development but peaked during mid-development, which is thought to reflect changes in volatile substances [62]. This indicates that *LOX* genes are significant in regulating the synthesis of volatile substances and are related to the degree of fruit development.

## 5. Conclusions

In this study, 12 bitter gourd *LOX* gene family members were identified and obtained. Bioinformatics methods were used to analyze the distribution, chromosomal localization, gene structure, evolutionary affinities and cis-acting elements of the *LOX* gene in the whole genome of bitter gourd. An analysis of *McLOX* gene expression by RT-qPCR indicated that *McLOX* gene family members were expressed differently in tissue. The determination of monoterpene volatiles in bitter gourd of different fruit colors by GC-MS showed that the “green” flavor of bitter gourd mainly originated from alcohols. This study further elucidated the impact of the *LOX* gene in the lipoxygenase pathway of bitter gourd.

## Figures and Tables

**Table 1:** Physico-chemical properties of McLOX.

Gene Name.	Gene ID	Amino Acid	Molecular Weight (kDa)	PI	Gravy	Instability Index	Subcellular Localization
*McLOX1*	MC02g0081	763	86.74	5.43	−0.395	37.82	Cytoplasm
*McLOX2*	MC02g0083	902	102.56	6.03	−0.466	41.39	Cytoplasm
*McLOX3*	MC07g0692	958	109.65	7.18	−0.362	42.88	Cytoplasm
*McLOX4*	MC08g2254	895	101.42	6.18	−0.33	40.53	Cytoplasm
*McLOX5*	MC08g2255	873	98.88	6.05	−0.345	39.7	Cytoplasm
*McLOX6*	MC08g2256	880	99.67	6.02	−0.365	41.88	Cytoplasm
*McLOX7*	MC08g_new0589	859	99.19	6.21	−0.539	46.15	Cytoplasm
*McLOX8*	MC08g_new0591	862	97.52	5.85	−0.42	42.85	Cytoplasm
*McLOX9*	MC08g2257	796	91.07	5.38	−0.415	46.75	Cytoplasm
*McLOX10*	MC09g0182	910	102.96	6.91	−0.411	43.25	Cytoplasm
*McLOX11*	MC09g0795	827	94.55	8.46	−0.504	40.83	Cytoplasm
*McLOX12*	MC09g1854	926	104.91	7.19	−0.451	47.32	Cytoplasm

**Table 2:** Secondary structures of *McLOX*.

Gene Name	α-Helix/%	Extended Strand/%	β-Turn/%	Random Coil/%
*McLOX1*	31.59	19.92	9.83	38.66
*McLOX2*	34.04	18.07	9.2	38.69
*McLOX3*	38	16.18	9.19	36.64
*McLOX4*	39.44	16.42	9.16	34.97
*McLOX5*	36.08	18.21	10.08	35.62
*McLOX6*	35	17.16	10.57	37.27
*McLOX7*	39	16.65	8.03	36.32
*McLOX8*	31.79	18.21	11.14	38.86
*McLOX9*	37.56	15.58	10.93	35.93
*McLOX10*	36.15	16.26	8.68	38.9
*McLOX11*	38	15.72	8.46	37.73
*McLOX12*	34.99	18.79	9.18	37.04

**Table 3:** Protein domain/s identified in bitter gourd lipoxygenase (LOX) proteins (N→C).

Gene Name	Gene ID	PLAT/LH2 (IPR036392)	Lipoxygenase (IPR036226)	Iron Binding (IPR020833)
*McLOX1*	MC02g0081	2–73	74–763	416–430
*McLOX2*	MC02g0083	72–210	211–902	555–569
*McLOX3*	MC07g0692	118–258	259–958	609–623
*McLOX4*	MC08g2254	53–200	201–895	551–565
*McLOX5*	MC08g2255	39–183	184–873	529–543
*McLOX6*	MC08g2256	40–184	185–880	536–550
*McLOX7*	MC08g_new0589	17–163	164–859	515–529
*McLOX8*	MC08g_new0591	18–165	167–862	518–532
*McLOX9*	MC08g2257	2–100	101–796	452–466
*McLOX10*	MC09g0182	77–219	221–910	564–578
*McLOX11*	MC09g0795	17–139	140–827	478–492
*McLOX12*	MC09g1854	90–230	231–926	579–593

**Table 4:** Consensus sequence of predicted McLOX motifs in bitter gourd.

Gene Name	*E*-Value	Width	Sites	Best Possible Match
Motif1	8.40E-399	50	12	DAGYHQLISHWLNTHAVIEPFVIATNRQLSVMHPIYKLLHPHFRDTMNIN
Motif2	1.10E-333	50	12	DKKDEPWWPKMQTLQDLIESCTTIIWIASALHAAVNFGQYPYGGYVPNRP
Motif3	8.50E-322	50	12	ALPADLIKRGVAVEDPSSPHGLRLLIEDYPFAVDGLEIWSAIKTWVTDYC
Motif4	9.60E-287	50	12	IFFANKSYLPSETPEPLRKYREEELLNLRGBGKGERKEWDRIYDYDVYND
Motif5	5.10E-234	41	12	AWRTDEEFARZMLAGVNPVIIRRLQEFPPLSKLDPEIYGDQ
Motif6	7.90E-178	50	8	GIPGAFFIRNGHTSEFFLKSLTLEDVPGHGRIHFDCNSWVYPSRRYKKDR
Motif7	4.50E-152	31	12	GLTVDEAJKQNKLYILDHHDALMPYLRRINS
Motif8	2.70E-208	50	12	YKELESNPEKAFLRTJPSQLQALLGVSLIEILSRHSPDEVYLGQRASPEW
Motif9	1.50E-148	29	12	TKTYATRTLLFLKEDGTLKPLAIELSLPH
Motif10	4.50E-140	29	12	ALARQSLINADGIJESTHFPGKYSMELSS
Motif11	2.20E-139	36	12	LKEIEERIMRRNKDPRLKNRTGPVVVPYTLLFPSSS
Motif12	7.40E-124	41	11	IYVPRDERFGHLKMSDFLAYALKSLSHSJVPGLESLFDSTP
Motif13	6.40E-108	29	11	GAISKVYFPAEEGVESSIWQLAKAYVAVN
Motif14	1.80E-105	21	12	GGKZYPYPRRGRTGRPPSKKD
Motif15	6.30E-101	50	7	EFDKFQDVHDLYEGGFPVPLNLLENLTENIPPPLFKEJLRSDGERFLKFP

## Data Availability

The data presented in this study are available on request from the corresponding author.

## Supplementary Materials

The following supporting information can be downloaded at: https://www.mdpi.com/article/10.3390/genes15121557/s1, Table S1. Primer sequences used for RT-qPCR amplification of McLOX. Table S2. McLOX nucleotide sequences of bitter gourd. Table S3. McLOX protein sequences of bitter gourd.

## References

[1] Liavonchanka A., Feussner I. (2006). Lipoxygenases: Occurrence, functions and catalysis. J. Plant Physiol..

[2] Hildebrand D.F. (1989). Lipoxygenases. Physiol. Plant..

[3] Gigot C., Ongena M., Fauconnier M.L., Wathelet J.P., Du Jardin P., Thonart P. (2010). The lipoxygenase metabolic pathway in plants: Potential for industrial production of natural green leaf volatiles. BASE.

[4] Feussner I., Kühn H., Wasternack C. (2001). Lipoxygenase-dependent degradation of storage lipids. Trends Plant Sci..

[5] Acosta I.F., Laparra H., Romero S.P., Schmelz E., Hamberg M., Mottinger J.P., Moreno M.A., Dellaporta S.L. (2009). *tasselseed1* is a lipoxygenase affecting jasmonic acid signaling in sex determination of maize. Science.

[6] Chen G., Hackett R., Walker D., Taylor A., Lin Z., Grierson D. (2004). Identification of a specific isoform of tomato lipoxygenase (TomloxC) involved in the generation of fatty acid-derived flavor compounds. Plant Physiol..

[7] Chuck G. (2010). Molecular Mechanisms of Sex Determination in Monoecious and Dioecious Plants. Adv. Bot. Res..

[8] Kolomiets M.V., Hannapel D.J., Chen H., Tymeson M., Gladon R.J. (2001). Lipoxygenase is involved in the control of potato tuber development. Plant Cell.

[9] Chauvin A., Caldelari D., Wolfender J.L., Farmer E.E. (2013). Four 13-lipoxygenases contribute to rapid jasmonate synthesis in wounded *Arabidopsis thaliana* leaves: A role for lipoxygenase 6 in responses to long-distance wound signals. New Phytol..

[10] Sarde S.J., Bouwmeester K., Venegas-Molina J., David A., Boland W., Dicke M. (2019). Involvement of sweet pepper CaLOX2 in jasmonate-dependent induced defence against Western flower thrips. J. Integr. Plant Biol..

[11] Yang X.Y., Jiang W.J., Yu H.J. (2012). The expression profiling of the lipoxygenase (LOX) family genes during fruit development, abiotic stress and hormonal treatments in cucumber (*Cucumis sativus* L.). Int. J. Mol. Sci..

[12] Tsitsigiannis D.I., Keller N.P. (2007). Oxylipins as developmental and host-fungal communication signals. Trends Microbiol..

[13] Baldwin I.T., Schmelz E.A., Ohnmeiss T.E. (1994). Wound-induced changes in root and shoot jasmonic acid pools correlate with induced nicotine synthesis in*Nicotiana sylvestris* spegazzini and comes. J. Chem. Ecol..

[14] Leone A., Bleve-Zacheo T., Gerardi C., Melillo M.T., Leo L., Zacheo G. (2006). Lipoxygenase involvement in ripening strawberry. J. Agric. Food Chem..

[15] Chang-Rong G., Yan-Mei L.I., Li-Jun Y.J. (2003). Relationship Between LOX Activity, SA and JA Accumulation in Tobacco Leaves Under Water Stress. Agric. Sci. China.

[16] Vogt J., Schiller D., Ulrich D., Schwab W., Dunemann F. (2013). Identification of lipoxygenase (LOX) genes putatively involved in fruit flavour formation in apple (Malus × domestica). Tree Genet. Genomes.

[17] Zhang C., Jin Y., Liu J., Tang Y., Cao S., Qi H.J.S.H. (2014). The phylogeny and expression profiles of the lipoxygenase (LOX) family genes in the melon (*Cucumis melo* L.) genome. Sci. Hortic..

[18] Zhang B., Chen K., Bowen J., Allan A., Espley R., Karunairetnam S., Ferguson I. (2006). Differential expression within the LOX gene family in ripening kiwifruit. J. Exp. Bot..

[19] Cuevas-Glory L.F., Sosa-Moguel O., Pino J., Sauri-Duch E.J.F.A.M. (2015). GC–MS Characterization of Volatile Compounds in Habanero Pepper (*Capsicum chinense* Jacq.) by Optimization of Headspace Solid-Phase Microextraction Conditions. Food Anal. Methods.

[20] Vogel J.T., Tieman D.M., Sims C.A., Odabasi A.Z., Clark D.G., Klee H.J. (2010). Carotenoid content impacts flavor acceptability in tomato (*Solanum lycopersicum*). J. Sci. Food Agric..

[21] Trierweiler B., Frechen M.A., Soukup S.T., Egert B., Baldermann S., Sanguansil S., Mccreight J.D., Kulling S.E., Dhillon N.P.S. (2019). Bitter gourd, *Momordica charantia* L.; breeding lines differ in secondary metabolite content according to market type. J. Appl. Bot. Food Qual..

[22] Crops G.S.L.B.S.M.F.I.o.V., Shanghai F.J.A.A. (2004). Correlation and Principal Component Analyses on Major Agronomic Characters of Balsam Pear (*Momordica charantia* L.). Acta Agric. Shanghai.

[23] Yu-Sheng L.U., Zhi-Xiong L., Ji-Shui Q., Xiao-Xiao C., Jian-Ping P.J.A.H.S. (2016). Fruit Character Diversity Analysis and Numerical Taxonomy of Wampee (*Clausena lansium*) Germplasm Resources. Acta Hortic. Sin..

[24] Chambers E.t., Koppel K. (2013). Associations of volatile compounds with sensory aroma and flavor: The complex nature of flavor. Molecules.

[25] Tieman D., Bliss P., McIntyre L.M., Blandon-Ubeda A., Bies D., Odabasi A.Z., Rodríguez G.R., van der Knaap E., Taylor M.G., Goulet C. (2012). The chemical interactions underlying tomato flavor preferences. Curr. Biol. CB.

[26] Min Y. (2010). SPME-GC-MS Analysis of Volatile Composition of Bitter Melon Fruits. Food Sci..

[27] Finn R.D., Clements J., Eddy S.R. (2011). HMMER web server: Interactive sequence similarity searching. Nucleic Acids Res..

[28] Chen C., Chen H., Zhang Y., Thomas H.R., Frank M.H., He Y., Xia R. (2020). TBtools: An Integrative Toolkit Developed for Interactive Analyses of Big Biological Data. Mol. Plant.

[29] Wenli D.U., Zhongshan C., Duanxiang X.U., Tongwei X.U., Shan G.A.O., Qingfang W.E.N. (2021). Physiological Response and Differentially Expressed Genes Analysis of Transcriptome in *Momordica charantia* L. Leaf Under Cold Stress. J. Nucl. Agric. Sci..

[30] Arocho A., Chen B., Ladanyi M., Pan Q. (2006). Validation of the 2-DeltaDeltaCt calculation as an alternate method of data analysis for quantitative PCR of BCR-ABL P210 transcripts. Diagn. Mol. Pathol..

[31] Djavanshir D., Abolghasem J., Parastou M. (2017). Volatile Organic Compounds Trapping from Gaseous Samples on the Basis of Co-Liquefaction with Organic Solvent for Gas Chromatographic Analysis. Curr. Anal. Chem..

[32] Bai J., Baldwin E.A., Imahori Y., Kostenyuk I., Burns J., Brecht J.K. (2011). Chilling and heating may regulate C6 volatile aroma production by different mechanisms in tomato (*Solanum lycopersicum*) fruit. Postharvest Biol. Technol..

[33] Ties P., Barringer S. (2012). Influence of lipid content and lipoxygenase on flavor volatiles in the tomato peel and flesh. J. Food Sci..

[34] Buescher R.H., Buescher R.W.J.J.o.F.S. (2010). Production and Stability of (E, Z)-2, 6-Nonadienal, the Major Flavor Volatile of Cucumbers. J. Food Sci..

[35] Echeverria G., Graell J., Lopez M.L., Lara I.J.P.b. (2004). Volatile production, quality and aroma-related enzyme activities during maturation of ‘Fuji’ apples. Postharvest Biol. Technol..

[36] Pérez A.G., Sanz C., Olías R., Olías J.M. (1999). Lipoxygenase and Hydroperoxide Lyase Activities in Ripening Strawberry Fruits. Agric. Food Chem..

[37] Wu M., Chen K.S., Zhang S.L.J.A.H.S. (1999). Involvement of lipoxygenase in the postharvest ripening of peach fruit. Acta Hortic. Sin..

[38] Oliveira I., Guedes de Pinho P., Malheiro R., Baptista P., Pereira J.A. (2011). Volatile profile of *Arbutus unedo* L. fruits through ripening stage. Food Chem..

[39] Bannenberg G., Martínez M., Hamberg M., Castresana C. (2009). Diversity of the enzymatic activity in the lipoxygenase gene family of *Arabidopsis thaliana*. Lipids.

[40] Bell E., Creelman R.A., Mullet J.E. (1995). A chloroplast lipoxygenase is required for wound-induced jasmonic acid accumulation in Arabidopsis. Proc. Natl. Acad. Sci. USA.

[41] Melan M.A., Dong X., Endara M.E., Davis K.R., Ausubel F.M., Peterman T.K. (1993). An Arabidopsis thaliana lipoxygenase gene can be induced by pathogens, abscisic acid, and methyl jasmonate. Plant Physiol..

[42] Zhang J., Ng C., Jiang Y., Wang X., Wang S., Wang S. (2022). Genome-wide identification and analysis of LOX genes in soybean cultivar “Zhonghuang 13”. Front. Genet..

[43] Blee E., Joyard J. (1996). Envelope Membranes from Spinach Chloroplasts Are a Site of Metabolism of Fatty Acid Hydroperoxides. Plant Physiol..

[44] Feussner I., Wasternack C. (2002). The lipoxygenase pathway. Annu. Rev. Plant Biol..

[45] Dong W., Jiao B., Wang J., Sun L., Li S., Wu Z., Gao J., Zhou S. (2023). Genome-Wide Identification and Expression Analysis of Lipoxygenase Genes in Rose (*Rosa chinensis*). Genes.

[46] Wolters H., Jürgens G. (2009). Survival of the flexible: Hormonal growth control and adaptation in plant development. Nat. Rev. Genet..

[47] D’Onofrio C., Matarese F., Cuzzola A. (2018). Effect of methyl jasmonate on the aroma of Sangiovese grapes and wines. Food Chem.

[48] Li S.-t., Zhang M., Fu C.-h., Xie S., Zhang Y., Yu L.-j. (2012). Molecular Cloning and Characterization of Two 9-Lipoxygenase Genes from *Taxus chinensis*. Plant Mol. Biol. Report..

[49] Park Y.S., Kunze S., Ni X., Feussner I., Kolomiets M.V. (2010). Comparative molecular and biochemical characterization of segmentally duplicated 9-lipoxygenase genes ZmLOX4 and ZmLOX5 of maize. Planta.

[50] De La Fuente G.N., Murray S.C., Isakeit T., Park Y.S., Yan Y., Warburton M.L., Kolomiets M.V. (2013). Characterization of genetic diversity and linkage disequilibrium of ZmLOX4 and ZmLOX5 loci in maize. PLoS ONE.

[51] Gao X., Starr J., Göbel C., Engelberth J., Feussner I., Tumlinson J., Kolomiets M. (2008). Maize 9-lipoxygenase ZmLOX3 controls development, root-specific expression of defense genes, and resistance to root-knot nematodes. Mol. Plant-Microbe Interact. MPMI.

[52] Nemchenko A., Kunze S., Feussner I., Kolomiets M. (2006). Duplicate maize 13-lipoxygenase genes are differentially regulated by circadian rhythm, cold stress, wounding, pathogen infection, and hormonal treatments. J. Exp. Bot..

[53] Christensen S.A., Nemchenko A., Borrego E., Murray I., Sobhy I.S., Bosak L., DeBlasio S., Erb M., Robert C.A., Vaughn K.A. (2013). The maize lipoxygenase, ZmLOX10, mediates green leaf volatile, jasmonate and herbivore-induced plant volatile production for defense against insect attack. Plant J. Cell Mol. Biol..

[54] Li D.P., Xu Y.F., Sun L.P., Liu L.X., Hu X.L., Li D.Q., Shu H.R. (2006). Salicylic acid, ethephon, and methyl jasmonate enhance ester regeneration in 1-MCP-treated apple fruit after long-term cold storage. J. Agric. Food Chem..

[55] Yang C., Wang Y., Wu B., Fang J., Li S. (2011). Volatile compounds evolution of three table grapes with different flavour during and after maturation. Food Chem..

[56] Eduardo I., Chietera G., Bassi D., Rossini L., Vecchietti A. (2010). Identification of key odor volatile compounds in the essential oil of nine peach accessions. J. Sci. Food Agric..

[57] Pichersky E., Noel J.P., Dudareva N. (2006). Biosynthesis of plant volatiles: Nature’s diversity and ingenuity. Science.

[58] Tsao R., Yu Q. (2000). Nematicidal Activity of Monoterpenoid Compounds against Economically Important Nematodes in Agriculture. J. Essent. Oil Res..

[59] Wu S.H., Wu D.G., Chen Y.W. (2010). Chemical constituents and bioactivities of plants from the genus Paeonia. Chem. Biodivers..

[60] Zhang B., Shen J.Y., Wei W.W., Xi W.P., Xu C.J., Ferguson I., Chen K. (2010). Expression of genes associated with aroma formation derived from the fatty acid pathway during peach fruit ripening. J. Agric. Food Chem..

[61] Zhang B., Yin X.R., Li X., Yang S.L., Ferguson I.B., Chen K.S. (2009). Lipoxygenase gene expression in ripening kiwifruit in relation to ethylene and aroma production. J. Agric. Food Chem..

[62] Li P.C., Yu S.W., Shen J., Li Q.Q., Li D.P., Li D.Q., Zheng C.C., Shu H.R. (2014). The transcriptional response of apple alcohol acyltransferase (MdAAT2) to salicylic acid and ethylene is mediated through two apple MYB TFs in transgenic tobacco. Plant Mol. Biol..

